# Double Inferior Vena Cava With Mega Cava: A Case Report

**DOI:** 10.7759/cureus.82639

**Published:** 2025-04-20

**Authors:** Michael J Sinnott, David Kosoy, Albert Xing

**Affiliations:** 1 Medicine, Florida International University (FIU) Herbert Wertheim College of Medicine, Tampa, USA; 2 Radiology, Aventura Hospital and Medical Center, Aventura, USA; 3 Interventional Radiology, Aventura Hospital and Medical Center, Aventura, USA

**Keywords:** congenital venous anomaly, double inferior vena cava, endovascular management, ivc filter placement, mega cava

## Abstract

We present the case of a 68-year-old male with a history of hypertension, prostate cancer, and glioblastoma, who presented for inferior vena cava (IVC) filter placement due to a deep venous thrombosis (DVT) with elevated thromboembolic risk and a contraindication to anticoagulation secondary to fall risk from ataxia. Pre-procedural imaging revealed a rare double IVC variant with a mega cava configuration above the confluence - a condition that can complicate vascular interventions and increase thromboembolic events. The anatomical variation made a single IVC filter infeasible due to size constraints and instead prompted the use of dual IVC filters, one in each IVC, to ensure protection against pulmonary embolism. A double IVC occurs when there is persistence of the bilateral supracardinal veins during embryogenesis. In cases of mega cava (diameter >28 mm), conventional IVC filters are ineffective.

This case involved the placement of two filters to optimize protection against pulmonary embolism due to the large IVC diameter, with the option of a Bird's Nest filter as an alternative approach. Dual filters were chosen, as they effectively covered both venous channels while offering retrievability, compared to the Bird's Nest filter, which is permanent and may pose a higher risk of complications, including migration or IVC perforation. This case adds to the current knowledge base by demonstrating that dual IVC filter placement is an effective strategy for managing patients with a double IVC and mega cava when single filter placement is not possible.

This case highlights the importance of recognizing and managing unique anatomical variations in IVC filter placement. Endovascular surgery requires a strategic approach, utilizing pre-procedural imaging to plan the next steps and optimize thromboembolic protection.

## Introduction

Inferior vena cava (IVC) is the largest vein in the body and is responsible for returning deoxygenated blood from the lower extremities, kidneys, pelvis, and abdominal viscera to the right atrium of the heart [1,2]. A double IVC is a rare congenital anomaly, formed by the persistence of the supracardinal veins, and has an incidence of 0.2%-3% [3]. This variant can have significant clinical implications, especially in the context of thromboembolic disease. Studies have shown that a double IVC is associated with a greater risk of developing deep venous thrombosis (DVT) and increases the risk for venous thromboembolism [4]. This is due to altered hemodynamics, including diminished venous return and subsequent venous stasis, both of which facilitate thrombogenesis [4].

Anticoagulation may be contraindicated in cases such as recent major bleeding, intracranial hemorrhage, or severe bleeding diathesis [5]. If anticoagulation is contraindicated, management may require the use of IVC filters to prevent pulmonary embolism [6]. Management of patients with double IVC includes several options, such as a single filter placement above the confluence of the duplicated IVCs, dual filter placement with one in each venous channel, or a Bird's Nest filter for large cava configurations [7,8]. Newer technologies, such as retrievable and convertible IVC filters, allow for removal of the device when the risk of thromboembolism has decreased, which reduces long-term complications such as device migration and IVC perforation associated with permanent filters [9].

This case report presents a rare anatomical variant of a double IVC, highlighting the implications for endovascular procedures with a double IVC filter. The choice of treatment for double IVC filter placement is individualized based on the patient's anatomy and clinical scenario. This case aims to address the gap in the literature by demonstrating the challenges of selecting an appropriate IVC filter in patients with a double IVC and mega cava, where standard approaches are not feasible.

## Case presentation

A 68-year-old male with a past medical history of hypertension, prostate cancer status post surgery in 2022, and glioblastoma WHO Grade IV presented for IVC filter placement. His prostate cancer was in remission, and his glioblastoma remained active, which was the primary factor driving his prognosis and risk of thrombosis. Due to his multiple malignancies and immobility, he had an increased risk of DVT. An ultrasound revealed a positive DVT of the left popliteal vein, which put him at risk for a potential pulmonary embolism and prompted prophylactic treatment. Anticoagulation was contraindicated in this patient due to severe ataxia secondary to glioblastoma, which placed him at high risk of falls and intracranial hemorrhage [5]. This clinical context elicited the use of mechanical prophylaxis with an IVC filter.

**Figure 1:**
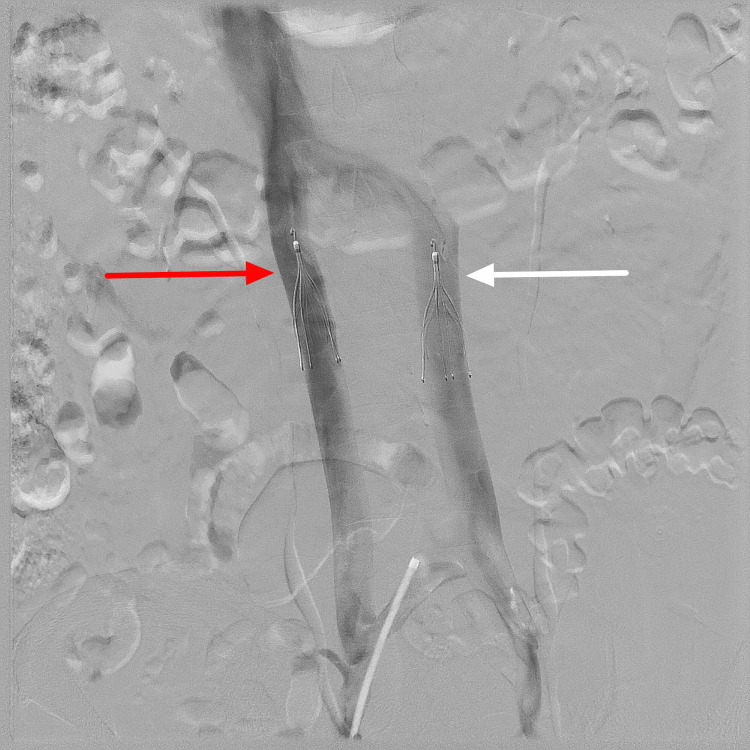
Angiogram demonstrates successful placement of a filter in the right IVC (red arrow) and the left IVC (white arrow). IVC, Inferior vena cava

**Figure 2:**
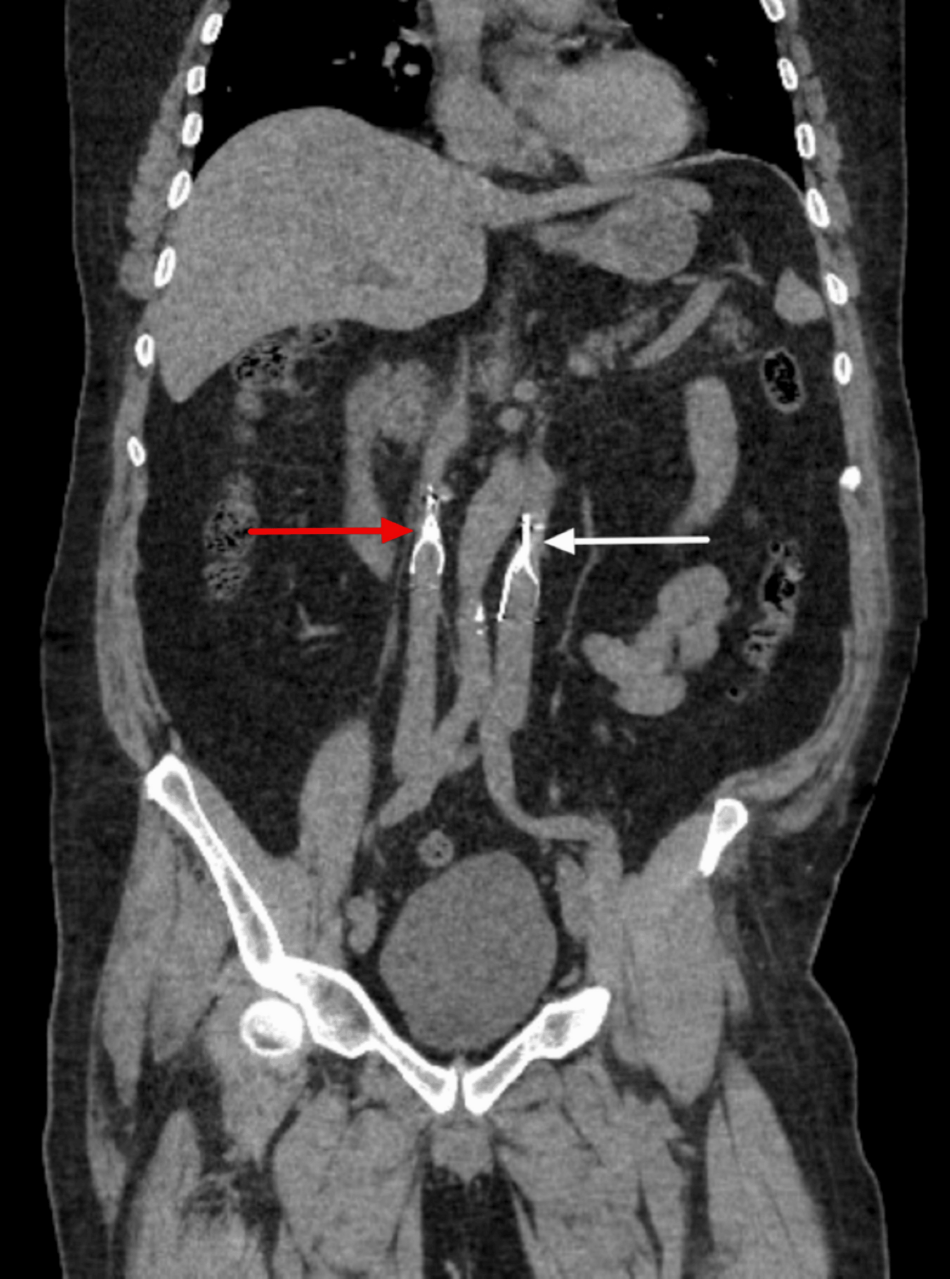
Coronal CT demonstrates a filter in the right IVC (red arrow) and the left IVC (white arrow). CT, Computed tomography; IVC, Inferior vena cava

**Figure 3:**
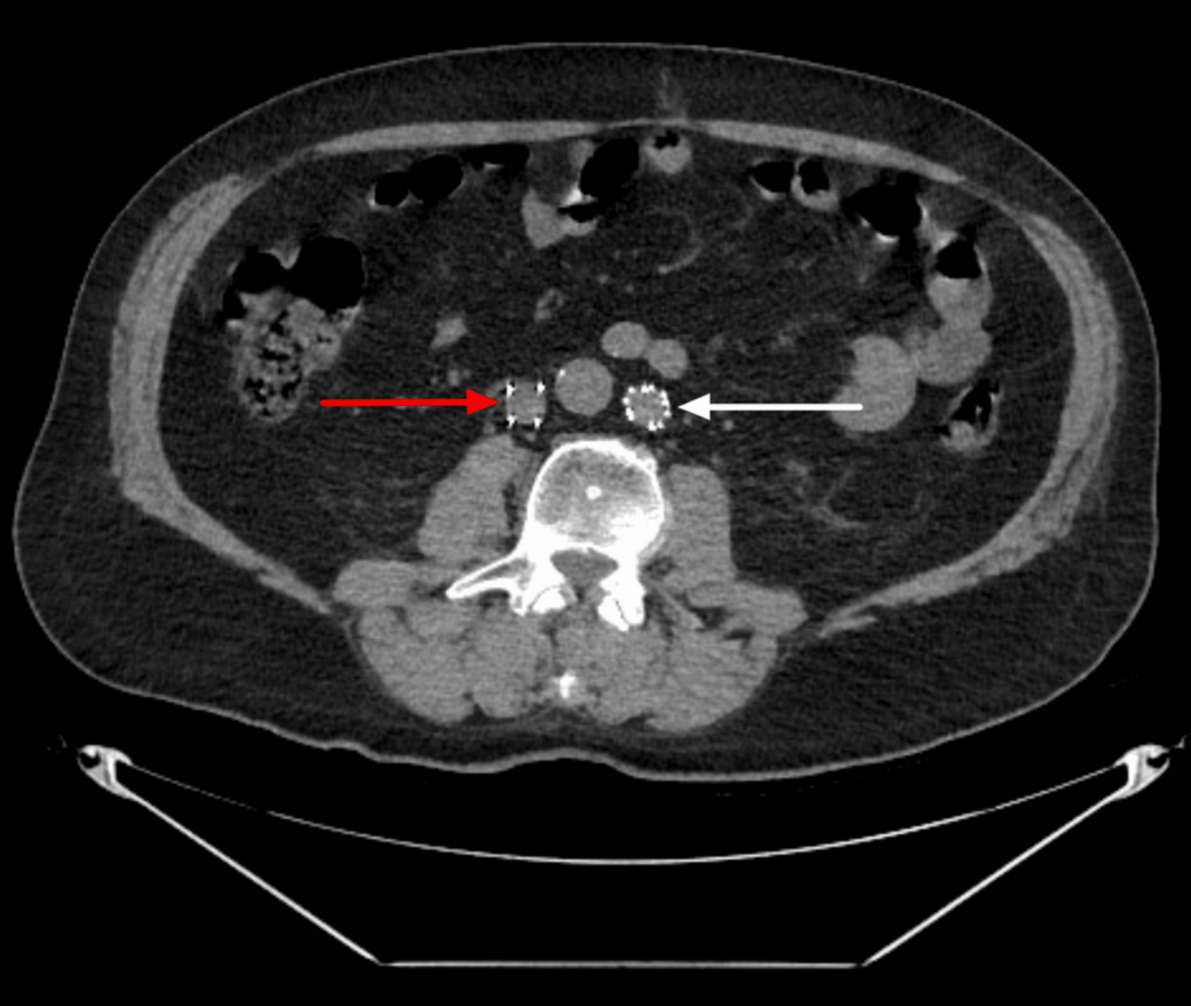
Axial CT demonstrates a filter in the right IVC (red arrow) and the left IVC (white arrow). CT, Computed tomography; IVC, Inferior vena cava

Pre-procedural imaging with computed tomography (CT) was performed as part of the institution's standard protocol to assess IVC anatomy before filter placement. There was no prior suspicion of the patient having an anatomical variant, and the duplicated IVC was found incidentally, with a presumed mega cava configuration, defined as an IVC diameter >28 mm [10]. The patient's IVC diameter was measured to be 41 mm, which presented a unique challenge, as a single IVC filter placement could no longer be performed since conventional filters are designed for diameters up to 28 mm. Given the patient's anatomical complexity, it was determined that dual filter placement - one filter in each IVC - would provide optimal protection against pulmonary embolism (Figures 1-3). The procedure was performed in the catheterization room without any complications. Right internal jugular vein access was chosen due to its anatomical advantage in providing easier access to the IVC. Celect Platinum Vena Cava Filters (maximum filter diameter = 30 mm, filter length = 49 mm, introducer sheath = 7.0 French) were selected for deployment. The patient tolerated the procedure well and was closely monitored afterward, with no concerning findings noted. At the time of the procedure, there was no short-term plan for filter retrieval. The patient was scheduled for a three-month follow-up to assess the need for continued filter use.

## Discussion

The IVC develops between the fourth and eighth week from three embryonic venous structures: the posterior cardinal veins, subcardinal veins, and supracardinal veins [11]. During normal development, the posterior cardinal veins regress, while the subcardinal veins contribute to the suprarenal portion of the IVC, and the supracardinal veins develop the infrarenal segment [11]. In most cases, the left-sided venous structures regress, allowing for only a single right-sided IVC. However, in some cases, there can be persistence of both supracardinal veins in the infrarenal region, which prevents fusion into a single venous trunk and instead leads to a duplicated IVC [11]. Typically, the right IVC follows a normal anatomical course, while the left IVC crosses anterior to the abdominal aorta and joins with the right IVC at the level of the renal veins [12]. In cases where the subcardinal veins fail to fuse, the duplication extends to the suprarenal portion [12].

These embryologic variations are clinically significant, as they increase the risk of DVT due to altered venous drainage and increased blood stasis [4]. A double IVC is usually asymptomatic and is found incidentally, such as through imaging or during surgical procedures. The presence of a duplicated IVC alters vascular access and may complicate IVC filter placement, increasing the risk of misplacing the device if the anomaly is not recognized. During retroperitoneal procedures, there is a greater risk of vascular injury, such as during a nephrectomy or adrenalectomy [12]. In some cases, a duplicated IVC can even be mistaken for other pathological findings, such as lymphadenopathy on imaging, leading to unnecessary interventions [13]. Being aware of these variations is crucial prior to abdominal and pelvic surgical interventions in order to prevent misdiagnosis and ensure appropriate treatment.

IVC filters are devices implanted into the IVC that prevent the formation of a pulmonary embolism by stopping thrombi that form in the lower extremities [14]. IVC filters are typically used in patients who have a contraindication to anticoagulation or those who have failed anticoagulation therapy [6,14]. Placement of IVC filters is a technically challenging procedure, with a risk of complications such as filter migration, thrombosis, and difficulty with retrieval [15]. Filters also increase the risk of lower extremity DVT, which may be due to emboli entrapment in the filter, DVT extension from the lower extremities, or thrombogenicity of the device [16]. There are several approaches to utilizing IVC filters for a patient with double IVC, with the most common options being single filter placement and dual filter placement. Current literature does not provide evidence as to the DVT rates between the placement of one versus two IVC filters.

In single filter placement, a filter is placed above the confluence of the duplicated IVCs and effectively prevents thromboembolism from either venous channel [15]. In some cases, such as this, a single filter placement is not possible. Because most standard IVC filters are only made for IVCs 28 mm or less, the use of a Bird’s Nest filter can be implemented, as it can be used for IVCs up to 40 mm [8]. The use of a Bird's Nest filter was ruled out due to the risks associated with using permanent filters, such as migration and IVC wall perforation [9]. Another option is to use double IVC filters, allowing for the placement of two filters, one in each limb of the duplicated IVC, ensuring protection against both venous channels [17]. Potential risks of using a standard IVC filter include increased risk of thrombosis, filter migration, and vessel perforation, but it has a smaller risk compared to permanent filters, such as the Bird's Nest filter. Currently, there is no preference for using a single filter compared to a double filter when treating a double IVC.

In this case, the patient had an increased risk of thromboembolism due to his DVT and history of malignancies, but was unable to be put on anticoagulants due to his fall risk from glioblastoma-related ataxia. This necessitated the placement of an IVC filter, but further imaging revealed a double IVC and a mega cava - an anomaly that occurs in less than 1% of the population [10,18]. The coexistence of a duplicated IVC and mega cava in a single patient is extremely rare, and no other cases were found in the literature. The presence of a mega cava made the use of a single-filter placement insufficient for pulmonary embolism prevention, as the large size of the cava could not accommodate a standard IVC filter. These anatomical challenges were addressed with two filters, strategically placed within both IVC vessels. The decision for two filters was guided by the need to cover both venous channels to prevent thrombus bypass and embolization, which would not have been as effectively covered with the use of a single filter. Retrievable filters were chosen, as they allow the option to remove them in the future if the patient's condition improves. The patient is scheduled for follow-up imaging to assess each filter's position and identify any complications.

This case adds to existing knowledge by highlighting challenges and demonstrating effective management of double IVC with the use of two retrievable filters in a patient with a rare anatomical variation. It also adds to the decision-making frameworks by showcasing the use of dual IVC filters as a viable option when a single filter is unfavorable. This expands the understanding of how to manage complex venous anomalies, with an increased risk of thromboembolism, in patients who are unable to use anticoagulation.

## Conclusions

This case demonstrates the challenges of IVC filter placement in patients with anatomical abnormalities, such as double IVC and mega cava. Double IVC is an anomaly that is rare but clinically significant, as it may complicate vascular interventions and require careful procedural imaging and management. A coexisting mega cava further complicated the case by making the use of a single filter infeasible. Two standard filters were successfully used to address the patient's unique anatomy and reduce the thromboembolic risk. Being aware of anatomical variations is crucial to ensure optimal patient outcomes and avoid complications during surgery. Limitations of this case include the absence of long-term follow-up data and limited generalizability, due to the rarity of this variant. This case emphasizes the need for procedural planning and preoperative imaging to identify and tailor an appropriate treatment plan. Additionally, multidisciplinary decision-making with radiology, vascular surgery, and oncology teams is essential for managing complex anomalies such as double IVC and promoting a comprehensive treatment plan. Creating guidelines and implementing protocols for managing double IVC and mega cava cases can ensure safer interventions and optimize outcomes in the future.
